# The Impact of Spatial Navigation Task Types: Moderator Analysis in Meta‐Analysis on Mild Cognitive Impairment

**DOI:** 10.1002/alz.091626

**Published:** 2025-01-03

**Authors:** Dorota Kossowska‐Kuhn, Gillian Gouveia, Neil Charness

**Affiliations:** ^1^ Florida State University, Tallahassee, FL USA; ^2^ Florida State University, tallahassee, FL USA

## Abstract

**Background:**

Dementia exerts a significant global impact on societies and individuals. Spatial disorientation emerges as one of the initial symptoms of Alzheimer’s Disease (AD) (Coughlan et al., 2018). In our Meta‐analysis on spatial navigation in Mild Cognitive Impairment (MCI) we obtained the standardized mean difference (Hedge’s g) between this population and cognitively healthy older adults at the level of g = .88, which corresponds to the large effect size.

**Method:**

The potential moderators such as year of study publication, country of study, age of participants in each population, gender, education, forms of test administration (i.e., real‐world, virtual reality), type of measure (time, accuracy), type of task (i.e., A Maze, Environment Navigation, Money Road Map Test) were coded for moderator analysis.

**Result:**

Moderator analysis for 125 effect sizes across 47 studies, involving 2916 participants (1415 with MCI, 1501 cognitively healthy older adults) revealed that both age and the type of task were statistically significant (p < .05).

**Conclusion:**

Further discussion on the definition of spatial navigation skills is essential to reach a consensus regarding the tasks used for assessments. Given the potential role of spatial navigation as an early marker of dementia, the significance of understanding this complex skill is paramount.

